# Chronic infarct size after spontaneous coronary artery dissection: implications for pathophysiology and clinical management

**DOI:** 10.1093/eurheartj/ehz895

**Published:** 2020-01-03

**Authors:** Abtehale Al-Hussaini, Ahmed M S E K Abdelaty, Gaurav S Gulsin, Jayanth R Arnold, Marcos Garcia-Guimaraes, Diluka Premawardhana, Charley Budgeon, Alice Wood, Nalin Natarajan, Kenneth Mangion, Roby Rakhit, Stephen P Hoole, Thomas W Johnson, Colin Berry, Ian Hudson, Anthony H Gershlick, Andrew Ladwiniec, Jan Kovac, Iain Squire, Nilesh J Samani, Sven Plein, Gerry P McCann, David Adlam

**Affiliations:** 1 Department of Cardiovascular Sciences, NIHR Leicester Biomedical Research Centre, University of Leicester, Glenfield Hospital, Groby Road, Leicester LE3 9QP, UK; 2 Department of Cardiovascular Sciences, Faculty of Medicine, Suez Canal University, km 4.5 Ring road, 41522, Ismailia, Egypt; 3 Department of Cardiology, Hospital Universitario de La Princesa Madrid, Calle Diego de León 62, Madrid, Spain; 4 British Heart Foundation Glasgow Cardiovascular Research Centre, Institute of Cardiovascular and Medical Sciences, University of Glasgow, 126 University Place, Glasgow G12 8TA, UK; 5 Golden Jubilee National Hospital, Agamemnon Street, Clydebank G81 4DY, UK; 6 Royal Free Hospital, Pond St, Hampstead, London NW3 2QG, UK; 7 Institute of Cardiovascular Science University College London, 62 Huntley St, Fitzrovia, London WC1E 6DD, UK; 8 Royal Papworth Hospital, Papworth Rd, Cambridge CB2 0AY, UK; 9 NIHR Cambridge Biomedical Research Centre, Box 277 Hills Road Cambridge CB2 0QQ, UK; 10 Department of Cardiology, Bristol Heart Institute, University Hospitals Bristol NHSFT & University of Bristol, Bristol BS2 8HW, UK; 11 Department of Biomedical Imaging Science, Leeds Institute of Cardiovascular and Metabolic Medicine, University of Leeds, Clarendon Way, Leeds LS2 9JT, UK

**Keywords:** Spontaneous coronary artery dissection, Cardiac magnetic resonance imaging

## Abstract

**Aims:**

To report the extent and distribution of myocardial injury and its impact on left ventricular systolic function with cardiac magnetic resonance imaging (CMR) following spontaneous coronary artery dissection (SCAD) and to investigate predictors of myocardial injury.

**Methods and results:**

One hundred and fifty-eight angiographically confirmed SCAD-survivors (98% female) were phenotyped by CMR and compared in a case–control study with 59 (97% female) healthy controls (44.5 ± 8.4 vs. 45.0 ± 9.1 years). Spontaneous coronary artery dissection presentation was with non-ST-elevation myocardial infarction in 95 (60.3%), ST-elevation myocardial infarction (STEMI) in 52 (32.7%), and cardiac arrest in 11 (6.9%). Left ventricular function in SCAD-survivors was generally well preserved with small reductions in ejection fraction (57 ± 7.2% vs. 60 ± 4.9%, *P* < 0.01) and increases in left ventricular dimensions (end-diastolic volume: 85 ± 14 mL/m^2^ vs. 80 ± 11 mL/m^2^, *P* < 0.05; end-systolic volume: 37 ± 11 mL/m^2^ vs. 32 ± 7 mL/m^2^, *P* <0.01) compared to healthy controls. Infarcts were small with few large infarcts (median 4.06%; range 0–30.9%) and 39% having no detectable late gadolinium enhancement (LGE). Female SCAD patients presenting with STEMI had similar sized infarcts to female Type-1 STEMI patients age <75 years. Multivariate modelling demonstrated STEMI at presentation, initial TIMI 0/1 flow, multivessel SCAD, and a Beighton score >4 were associated with larger infarcts [>10% left ventricular (LV) mass].

**Conclusion:**

The majority of patients presenting with SCAD have no or small infarctions and preserved ejection fraction. Patients presenting with STEMI, TIMI 0/1 flow, multivessel SCAD and those with features of connective tissue disorders are more likely to have larger infarcts.

**See page 2206 for the editorial comment on this article (doi: 10.1093/eurheartj/ehaa012)**

## Introduction

Spontaneous coronary artery dissection (SCAD) is a cause of acute myocardial infarction (AMI) and sudden cardiac death.^1^^,^^2^ It is characterized by the formation of a false lumen in the outer third of the coronary tunica media leading to external compression of the true lumen, coronary insufficiency and myocardial infarction.^3^ SCAD may be associated with true-luminal thrombus, particularly where there are fenestrations between true and false lumens.^4^ Although less common than atherosclerotic AMI, SCAD is not rare,^5^ particularly in young to middle-aged women, the primarily affected demographic group. A minority of cases (<10%) occur during or following pregnancy (P-SCAD) but these may constitute a more severe subgroup as an increase in ST-elevation myocardial infarction (STEMI) presentations and more proximal disease has been reported.^6^ The disease course following SCAD is not benign with significant rates of major adverse cardiovascular events (MACE) and SCAD recurrence reported (19.9% and 10.4% in a recent prospective observational series with median 3.1-year follow-up^7^).

Unlike for atherosclerotic acute coronary syndromes (ACS) where percutaneous coronary intervention (PCI) is recommended, current US Consensus and European Position Papers advocate a conservative approach to coronary revascularization where possible (for example, where TIMI flow is maintained or the affected coronary vessel subtends a modest myocardial territory).^1^^,^^2^ This is because conservatively managed SCAD appears to heal completely in most cases whilst PCI complications and suboptimal PCI outcome are common in reported series.

Cardiac magnetic resonance imaging (CMR) is the reference standard for *in vivo* quantification of LV function and myocardial infarction. The extent of myocardial infarction following SCAD using state-of-the-art CMR techniques has not been reported except in one small case series.^8^ We aimed to define the pattern of chronic infarction after SCAD using CMR. Factors influencing extent of infarction are reported and the implications both for the management of SCAD and for the pathophysiology of chronic infarction following SCAD-ACS are discussed.

## Methods

### Study population

The study was conducted in accordance with the Declaration of Helsinki. Consecutive patients and healthy controls were recruited from the UK Spontaneous Coronary Artery Dissection Registry to the SCAD Deep Phenotyping Study (ISRCTN42661582). Patients with SCAD in the registry are recruited from across the UK by self-referral, primary care physician referral and referral from the clinical team at the index presenting hospital. Healthy controls were recruited by open advertisement targeted to match the age/sex profile of the SCAD cohort. All participants provided fully informed signed consent to both the SCAD Registry and Deep Phenotyping Study. Comparator patients with atherosclerotic STEMI were consented female patients age <75 years undergoing follow-up CMR imaging following their index event. The protocol was approved by the UK National Research Ethics Service (14/EM/0056) and the UK Health Research Authority.

### Confirmation of spontaneous coronary artery dissection diagnosis

To be included in the UK SCAD registry, all patients must have had an angiographically confirmed diagnosis of SCAD. Patients with atherosclerotic, traumatic or iatrogenic dissection (except where the latter complicates definite SCAD) were excluded. Angiographic analysis was conducted by two experienced SCAD clinicians (A.A.-H. and M.G.G.) with adjudication of any differences by a third experienced SCAD clinician (D.A.). All angiographic analysis was conducted blinded to the results of the CMR analysis and images classified using a modified Yip and Saw^9^ classification. ST-elevation was defined as typical electrocardiography (ECG) changes occurring at any stage prior to angiography. Multivessel disease was defined as discontiguous SCAD locations affecting more than one coronary territory. Multisegment disease was defined as a single dissection site affecting more than one SYNTAX segment.^10^ Proximal disease was defined as dissections involving at least one of SYNTAX segments 1, 5, 6, or 11. PCI complications were defined as iatrogenic dissection; haematoma extension into a neighbouring SYNTAX segment; loss of flow in the stented vessel or a significant side branch (>2 mm diameter); and vessel perforation as shown by extravasation of contrast. Propensity-matched PCI vs. conservative managed cases were selected blinded to CMR findings and matched for STEMI/non-ST-elevation myocardial infarction (NSTEMI) status, TIMI flow, and angiographic appearance.

### Patient and spontaneous coronary artery dissection characteristics

Demographic information, medical history, and a detailed history of the SCAD event were obtained during the patient visit and clinical assessment including the measurement of a Beighton score (a simple nine-point clinical score to assess hypermobility and joint laxity^11^) recorded. Pregnancy-associated SCAD was defined as SCAD occurring during gestation or within 12 months of delivery.

### Cardiac magnetic resonance imaging acquisition and analysis

Spontaneous coronary artery dissection patients and healthy control CMR scanning was undertaken using a 3-T platform (Siemens Skyra, Erlangen, Germany). STEMI female controls were recruited from research studies from three UK centres (Leicester, Leeds, and Glasgow) having been imaged using similar protocols as previously published.^12–14^ Cine imaging with steady-state free precession and late gadolinium enhancement (LGE) imaging were performed in long-axis views and contiguous short-axis slices covering the entire left ventricle as previously described.^15^ LGE images were acquired 10–15 min after contrast administration [0.15 mmol/kg gadoterate meglumine (Dotarem), Guerbet Inc.] using a segmented inversion-recovery gradient-echo sequence. The inversion time was progressively adjusted to null unaffected myocardium.

Quantitative and qualitative analysis was performed offline blinded to patient details using Circle CVi42 software (Circle Cardiovascular Imaging, Calgary, Canada) by experienced observers (A.A.-H. and A.K.A.) with differences adjudicated by a third experienced reporter (G.P.M.). Left ventricular (LV) volumes and function were calculated as previously described.^16^ Infarct size was quantified semi-automatically on LGE imaging using the full-width half-maximum technique such that infarct size = (LGE mass/total LV mass)*100.^17^

### Statistical analysis

Summary statistics including counts and percentages are provided for categorical variables and both means and standard deviations and medians and interquartile ranges (IQR) are provided for continuous variables. Independent *t*-tests were performed to compare infarction in the SCAD patients to both the healthy control and STEMI controls.

Specifically for the female SCAD individuals, infarction was modelled in three ways. The correlates of the presence of any infarction (>0% compared to 0%) and large infarct size (10+% compared to <10%) were assessed using logistic regression. Odds ratios, 95% confidence intervals (CIs), and *P*-values are provided for all variables. For those who had measurable infarction (>0%), linear regression was performed to investigate the associations between potential correlates and the magnitude of infarction, when this was present on LGE. Two levels of adjustment were considered, one including and one excluding left mainstem (LMS). Univariately, means, 95% CIs, and *P*-values are provided for categorical variables. For the continuous variables and the multivariable results, estimated model coefficients (β), 95% CIs, and *P*-values are provided. Variables significant at the 5% level were retained in the final models. All statistical analyses were performed using the R environment for statistical computing.^18^

## Results

### Cohort demographics

One hundred and fifty-eight SCAD patients, 59 healthy controls, and 56 female Type-1 STEMI patients were recruited to the study. Subject characteristics are listed in *Table 1*. The SCAD cohort was predominantly female and ethnically Caucasian with a mean age at the time of SCAD of 45.8 years. Fifteen (9.5%) cases were P-SCAD. A third of SCAD patients presented with STEMI whilst 6.9% presented with primary ventricular arrhythmia leading to cardiac arrest.

**Table 1:** Summary of patient characteristics

	SCAD (*N* = 158)	HV (*N* = 59)	Type 1 STEMI (*N* = 56)
Female	155 (98.0%)	57 (96.6%)	56 (100%)
Age at SCAD (±SD) or age (years)	44.5 ± 8.4	45.0 ± 9.1	59.3 ± 9.8
BMI (±SD)	25.9 ± 5.6	25.9 ± 5.5	28.0 ± 7.4
Ethnicity			
Caucasian	149 (94.3%)	51 (87.9%)	52 (92.9%)
Indian	5 (3.1%)	3 (5.2%)	4 (7.1%)
Black African or Caribbean	1 (0.6%)	1 (1.7%)	0
Other	3 (1.9%)	2 (3.4%)	0
Smoking status			
Ever smoked	44 (28.3%)	21 (38.1%)	29 (51.8%)
Never smoked	114 (71.7%)	34 (61.8%)	26 (46.4%)
Unknown	0	0	1 (1.8%)
Medical history			
Diabetes	1 (0.6%)	0	13 (23.2%)
Hyperlipidaemia	12 (7.6%)	0	8 (14.3%)
Hypertension	30 (18.9%)	0	20 (35.7%)
Presentation			
STEMI	52 (32.7%)	NA	56 (100%)
NSTEMI	95 (60.3%)	NA	0
Cardiac arrest	11 (6.9%)	NA	0
Pregnancy status at time of SCAD			
Pregnant	3 (2.0%)	NA	0
Postpartum	12 (7.9%)	NA	0

### Angiographic findings and revascularization management

Angiographic findings are shown in Supplementary material online, *Table SA*. The left anterior descending was the most commonly affected coronary territory, involved in 68.6% of cases. Using the modified Yip–Saw SCAD classification, Type 2 SCAD was the commonest form (72.9%) with vessel occlusions (defined here as Type 4) present in 13.2% at angiography. Angiographically high-risk dissections were common with a proximal coronary segment involved in 20.1%, multivessel disease in 16.4%, and multisegment disease in 36.5%.

Fifty-six (35.8%) SCAD patients were managed with PCI with two-thirds of these managed with stenting. Of the patients managed with PCI 25 (44.6%) had a complication as a result of the procedure. The majority (20/25) were due to haematoma extension, with the remaining (5/25) cases due to distal coronary or branch occlusion resulting from PCI. A small number of patients (3.8%) were referred for emergency coronary artery bypass grafting (CABG), half after an initial attempt at PCI.

### Chronic infarction in spontaneous coronary artery dissection

**Figure 1:**
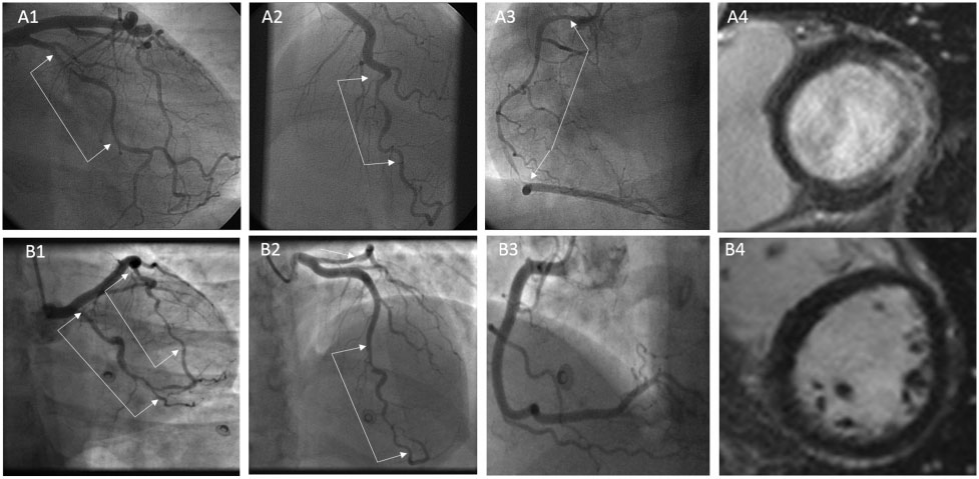
Exemplar images of spontaneous coronary artery dissection patients with varying infarct mass. Patient A had three-vessel spontaneous coronary artery dissection (*A1–A3*) leading to a large lateral infarct but sparing of the inferior, septal, and anterior walls (*A4*). Patient B had two-vessel spontaneous coronary artery dissection involving the left anterior descending and circumflex coronaries (*B1* and *B2*) but with no late gadolinium enhancement on cardiac magnetic resonance imaging (*B4*).

**Figure 2:**
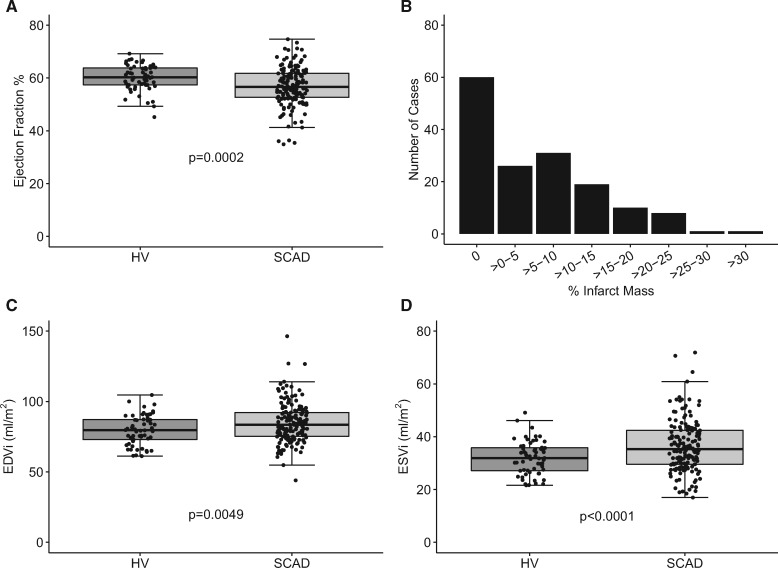
Impact of spontaneous coronary artery dissection on measures of left ventricular size and function. Effect of spontaneous coronary artery dissection on ejection fraction (*A*), body surface area adjusted left ventricular volumes (*C* and *D*), and the distribution of infarct sizes (*B*).

Patients with SCAD underwent follow-up CMR a median of 423 days (IQR 179–1045) following the index event. Eight patients were noted to have had recurrent SCAD. Exemplar images of patients with large and small infarcts coupled with coronary angiographic findings at the time of acute SCAD are shown in *Figure 1*. A qualitative variation in the pattern of infarction with relative sparing of the subendocardium was noted as a characteristic feature of SCAD (Supplementary material online, *Figure SA*). This pattern was observed in 11 SCAD cases (4 STEMI and 7 NSTEMI) but none of the atherosclerotic Type 1 STEMI controls. The extent of chronic infarction after SCAD is shown in *Figure 2*. Whilst there was a small but significant reduction in ejection fraction (*Figure 2A*; 56.8 ± 7.3% vs. 60.1 ± 4.8%, *P* = 0.0002) and an associated increase in end-diastolic (*Figure 2C*; 84.8 ± 14.2 mL/m^2^ vs. 79.7 ± 10.5 mL/m^2^, *P* = 0.0049) and end-systolic (*Figure 2D*; 37.1 ± 11.1 mL/m^2^ vs. 31.9 ± 6.3 mL/m^2^, *P* < 0.0001) volumes in SCAD compared to matched healthy controls, SCAD-infarcts were small (*Figure 2B*) with 60 (39%) patients having no demonstrable LGE (*Figure 2*) and only 6.4% having >20% infarct size.

The comparison of infarct characteristics due to STEMI in female SCAD-survivors with female patients <75 years with atherosclerotic Type 1 STEMI is shown in Supplementary material online, *Figure SB*. There was no significant difference in infarct size or end-systolic volume although female SCAD patients with STEMI had significantly higher ejection fractions (Supplementary material online, *Figure SBa*; 55.5 ± 7.2% vs. 50.9 ± 10.7%; *P* = 0.0094) and higher end-diastolic volume (Supplementary material online, *Figure SBc*; 90.2 ± 16.0 mL/m^2^ vs. 82.2 ± 20.6 mL/m^2^; *P* = 0.0260) compared to female Type 1 STEMI patients.

**Figure 3:**
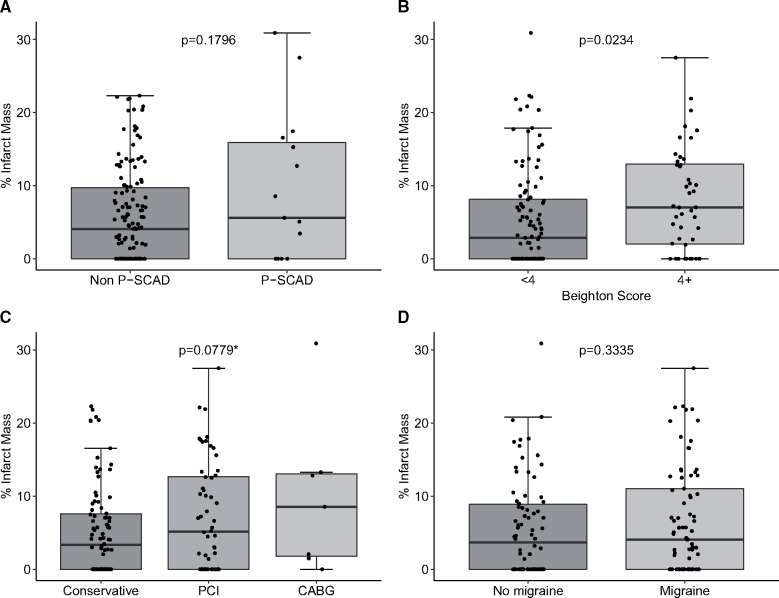
Effect of covariates on mean % infarct mass after spontaneous coronary artery dissection. Impact of pregnancy-associated spontaneous coronary artery dissection status (*A*), Beighton score (*B*), revascularization (*C*), and migraines (*D*). All *P*-values are from a *t*-test except when indicated with a * where analysis of variance was used.

The effect of a range of factors on infarct size is shown in *Figure 3* and Supplementary material online, *Figures SC* and *SD*.

### Predictors of infarction in spontaneous coronary artery dissection-survivors

#### Predictors of large infarcts (>10+% vs. ≤10%)

Univariate predictors of infarction >10% LV mass are shown in Supplementary material online, *Table SB* and multivariable predictors of presence of infarction are shown in *Table 2*. Univariately, increasing age at SCAD; left anterior descending SCAD; proximal vessel involvement; multivessel involvement; multisegment involvement; TIMI 0/1 vs. TIMI 2/3 flow; STEMI vs. NSTEMI presentation; and patients undergoing revascularisation compared to conservative management and patients with a Beighton score 4 compared to those with a score <4 were associated with >10% LV mass. Multivariably, STEMI presentation; initial TIMI flow 0/1 vs. 2/3; Beighton score 4+ vs. <4, and multivessel involvement were associated with infarction >10% of LV mass being present on LGE.

**Table 2:** Multivariable predictors of infarction in spontaneous coronary artery dissection survivors

Correlate	Infarction (>0% vs. 0%)			Infarction (10+% vs. 0 to <10%)		
	Univariate	Multivariable		Univariate	Multivariable	
	OR (95% CI)	OR (95% CI)	*P*-value	OR (95% CI)	OR (95% CI)	*P*-value
Initial TIMI flow						
TIMI 0/1	2.733 (1.149–6.497)	NS		3.014 (1.361–6.676)	3.272 (1.327–8.067)	0.01
TIMI 2/3	1			1	1	
Multivessel						
No	1	1	0.0336	1	1	0.0007
Yes	1.809 (0.708–4.619)	2.998 (1.089–8.252)		2.540 (1.049–6.152)	7.239 (2.310–22.685)	
STEMI/NSTEMI status						
NSTEMI	1	1	0.0079	1	1	0.0032
STEMI	2.786 (1.294–5.999)	3.651 (1.608–8.293)		3.547 (1.616–7.783)	5.189 (1.991–13.525)	
Cardiac arrest	1.714 (0.404–7.278)	1.924 (0.428–8.653)		2.533 (0.570–11.268)	3.108 (0.589–16.407)	
Beighton score						
<4	1	1	0.0059	1	1	0.0013
4+	2.543 (1.139–5.677)	3.37 (1.415–7.870)		2.556 (1.183–5.521)	4.741 (1.833–12.260)	

NS, not included in the final model; OR, odds ratio.

#### Predictors of any infarction (0% vs. >0%)

Univariate predictors of infarction >0% LV mass are shown in Supplementary material online, *Table SB* and multivariable predictors of presence of infarction are shown in *Table 2*. Univariately, TIMI 0/1 vs. TIMI 2/3 flow; STEMI vs. NSTEMI presentation; and patients with a Beighton score 4 compared to those with a score <4 were associated with >0% LV mass. Multivariably, STEMI presentation; Beighton score 4+ vs. <4 and multivessel involvement were associated with infarction >0% of LV mass being present on LGE.

#### Predictors of magnitude of infarction

Univariate predictors of magnitude of infarction in SCAD-survivors with measurable infarction (>0%) are shown in *Supplementary material online, Table SC* and multivariable predictors are shown in Supplementary material online, *Table SD*. Univariately, decreasing age, having the LMS vessel affected, having a recurrence and P-SCAD patients were all associated with increased infarct size. For the multivariable analysis, two levels of adjustment were considered; one including LMS and one excluding this covariate. When considering all variables, having the LMS vessel affected, multivessel involvement, recurrence, or presenting with STEMI, were independently associated with increasing magnitude of infarction. When excluding LMS, only recurrence and P-SCAD presentation were associated with increasing levels of infarction.

#### Propensity matching

No significant difference in infarct size was observed between 16 PCI and 16 conservative cases matched for ACS status (9 STEMI, 6 NSTEMI, and 1 cardiac arrest) TIMI flow (six TIMI 0/1 and 10 TIMI 2/3) and angiographic appearance (Supplementary material online, *Figure SE*; 5.9 ± 6.6% vs. 7.3 ± 7.1%; *P* = 0.5482).

## Discussion

We present the largest study to date to assess the impact of SCAD on chronic infarct size and left ventricular function using the reference test of CMR. We report, firstly that infarct sizes after SCAD are generally small and that large infarcts are rare. Secondly, that infarct size is increased in those with STEMI presentation, initial TIMI 0/1 flow at angiography, multivessel involvement and a high Beighton score (>4). Thirdly, that revascularization by PCI is not independently associated with reduced infarct size.

Although the initial presentation of SCAD is far from benign, with a third of patients presenting with STEMI and 6.9% with cardiac arrest, we have shown that a large proportion of patients (39%) have no detectable infarction and only a minority (6.4%) had very large infarction (>20%). Overall SCAD patients had a very small reduction in ejection fraction, but well within the normal range, in keeping with echocardiographic data which have suggested well preserved cardiac function in most cases.^19^ This is also consistent with data from observational cohort studies showing very low longer term mortality in SCAD-survivors.^2^^,^^20^

Modelling of covariates confirmed previous echocardiographic data that STEMI presentation is associated with increased chronic infarct size.^19^ In addition, multivessel involvement and reduced TIMI flow at angiography were, perhaps predictably, independently associated with larger infarcts. Hypermobility, as determined by a Beighton score >4, was found to predict chronic infarct size in SCAD-survivors. This is in keeping with the Canadian SCAD cohort study that reported connective tissue disease was an independent predictor of 30-day MACE in SCAD-survivors.^21^ Pregnancy-associated SCAD, although not an independent predictor of infarcts >10%, was associated with larger infarcts in the linear regression analysis of patients with measurable infarction once LMS was removed from the model. This multivariable modelling suggests an interaction between P-SCAD status and an increased risk of more proximal LMS dissections, suggesting it is the proximal nature of P-SCAD dissections which drives this adverse effect on infarct size. This is in keeping with previous reports from observational studies that P-SCAD is associated with an increased risk of proximal dissections and STEMI presentation.^6^

We provide novel insights into the role of revascularization in the management of SCAD. It is well-established that conservatively managed SCAD usually heals and that PCI is associated with high failure rates or suboptimal outcome, in part, due to extension of the false lumen due to tracking of the haematoma during stent deployment.^2^^,^^20^^,^^21^ Consistent with previous series, we also found high rates of complications in those patients undergoing PCI (25/56 or 45%; Supplementary material online, *Table SA*). The generally small infarcts observed suggest the management strategy adopted in these patients (2/3 managed conservatively) is associated with good outcomes from the perspective of left ventricular injury, albeit with some early hazard of dissection extension.^22^

However, this study also identifies higher risk SCAD cases such as those presenting with STEMI, poor TIMI flow or multivessel disease. Percutaneous coronary intervention was not independently associated with infarct size. This may be due, in part, to the fact that PCI complications did not appear to be associated with significantly increased chronic infarct size or to the confounding effect of other covariates such as STEMI presentation and TIMI flow interact and are themselves associated with adoption of PCI. Furthermore, in cases propensity matched for STEMI/NSTEMI presentation, TIMI flow, and angiographic appearance, there was no difference in % infarct mass between conservative and PCI managed cases. However, it should be noted that most of the cases included will be ‘grey zone’ cases, as those where management appears clear-cut will not be readily matched. Therefore, in the absence of randomized data, these data appear to support the currently recommended strategy of conservative management where possible in SCAD, reserving PCI for presentations at high risk of incurring large myocardial injuries (e.g. proximal vessel involvement with TIMI 0/1 flow and STEMI presentation). Such high-risk patients could form the basis for a future randomized controlled trial, perhaps with infarct size as the primary outcome, especially as we have shown no difference in infarct size in the STEMI cases vs. those with atherosclerotic type 1 STEMI.

Comparison of female Type 1 STEMI with SCAD STEMI suggests the different pathological aetiology underlying myocardial infarction in this population does not significantly impact on infarct size. One potential explanation is that this may result from balanced mechanisms with opposing effects on infarct size. For example, SCAD STEMI is associated with less true lumen thrombus than Type 1 STEMI.^4^ This might be expected to reduce infarction in SCAD STEMI and is a potential explanation for the relative endocardial sparing of LGE, we report in some SCAD infarcts. However, this could be balanced by the absence of a conditioning effect and collateralisation induced by a prior fixed stenosis (as found in Type 1 STEMI) which might tend to increase infarct size in the event of a SCAD-STEMI. There may also be differences in treatment delays or management between these groups. However, these findings do suggest that SCAD-STEMI patients, particularly those with poor TIMI flow, are at increased risk of larger infarcts akin to Type 1 STEMI, a finding which has implications for decisions about conservative vs. interventional management in this subpopulation of SCAD patients.

**Take home figure:**
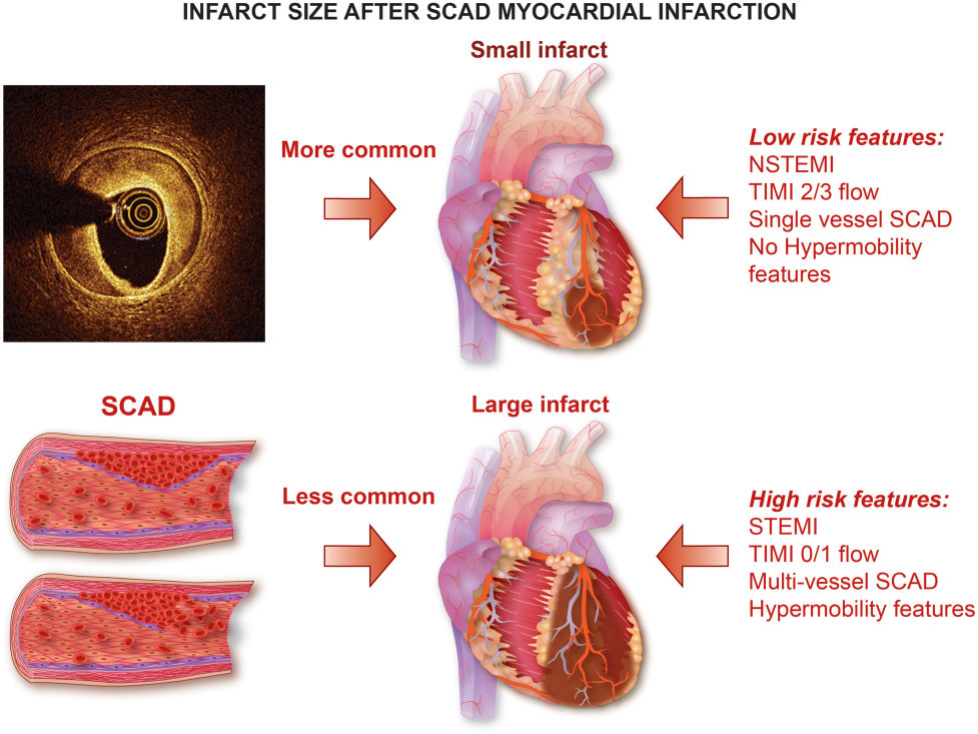
Infarct size after SCAR myocardial infarction.

### Limitations

This is an observational study and, therefore, we cannot conclude that the associations demonstrated are causative. Our data are derived from a SCAD-survivor cohort. Infarcts may be larger in those that died before assessment, although post-discharge mortality following SCAD is reportedly very low. Furthermore, the fact that patients recruited to the UK SCAD registry are retrospectively consented from national patient and clinician referrals may introduce selection bias. Although this might be expected to favour inclusion of more severely affected patients, there is also the potential that patients with a more severe clinical condition might not be prepared to consent to the rigorous phenotyping protocol. Although declined consent was not observed in practice, these factors have the potential to introduce bias. Additionally, because Type 1 atherosclerotic STEMI is uncommon in the demographic group affected by SCAD, it proved to be impossible to age-match the two groups. The comparison made should, therefore, be viewed in this context. The propensity-matched data are underpowered but are included to demonstrate a lack of any large differences in infarct size in equivalent cases managed by PCI or conservatively. Finally, our primary assessment in this study was with CMR, which is a surrogate of clinical outcome as infarct size is strongly related to subsequent clinical outcomes in myocardial infarction.^23^ The choice of 10% for larger infarcts is somewhat arbitrarily defined but represents above-average chronic infarct size in our lab following STEMI.^12^

## Conclusions

The majority of patients presenting with SCAD have no or small infarctions and preserved ejection fractions when assessed on follow-up CMR. Patients presenting with STEMI, reduced TIMI flow, multivessel SCAD, and those with features of connective tissue disorders are more likely to have bigger infarcts on CMR. PCI, while frequently associated with complications, appears not to induce larger infarcts.

## Supplementary Material

ehz895_Online_Supplementary_Data
