# Supplementary material for: Chronic infarct size after spontaneous coronary artery dissection: implications for pathophysiology and clinical management
Source: Eur Heart J. 2020 Jan 3;41(23):2197–205. doi: 10.1093/eurheartj/ehz895 (PMC7299635; doi:10.1093/eurheartj/ehz895)
Supplement: ehz895_Online_Supplementary_Data [file ehz895_online_supplementary_data.docx]

**Online supplement for: Myocardial injury after Spontaneous Coronary artery Dissection: implications for pathophysiology and clinical management**

**
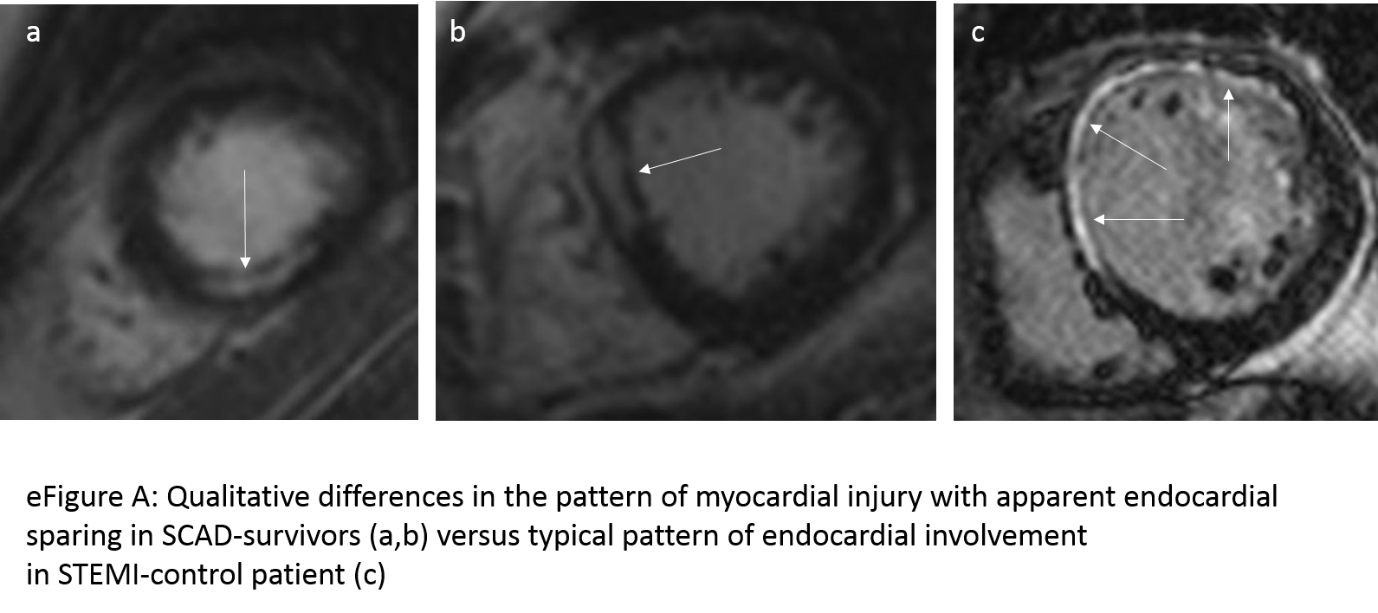
**

Figure SA: **Qualitative differences in the pattern of myocardial injury** with apparent endocardial

sparing in SCAD-survivors (a,b) versus typical pattern of endocardial involvement

in STEMI-control patient (c)

| **Table SA – Summary of angiogram features** | |
| --- | --- |
| **Vessel affected** |  |
| LMS | 10 (6.3%) |
| LAD | 109 (68.6%) |
| LCx | 51 (32.1%) |
| RCA | 25 (15.7%) |
| Proximal Segment Involved | 32 (20.1%) |
| Multi-vessel Involvement | 26 (16.4%) |
| Multi-segment Involvement | 58 (36.5%) |
| **SCAD angiographic classification** |  |
| Type 1 | 20 (12.6%) |
| Type 2a | 70 (44.0%) |
| Type 2b | 30 (18.9%) |
| Type 3 | 15 (9.4%) |
| Type 4 | 21 (13.2%) |
| **Mode of revascularisation** |  |
| Conservative | 95 (62.3%) |
| PCI | 56 (35.8%) |
| PCI complications | 25 (15.7) |
| CABG | 6 (3.8% - 3 after PCI) |
| **PCI (N=56)** |  |
| Wiring only | 3 (5.4%) |
| POBA only | 10 (17.9%) |
| Cutting balloon | 1 (1.8%) |
| Drug coated balloon | 1 (1.8%) |
| Stenting | 38 (67.9%) |
| **Stent type (N=38)** |  |
| BMS | 2 (5.3%) |
| DES | 33 (86.8%) |
| BVS | 3 (7.9%) |
| **Stent length mean (range)** | 53 (20-129) |
| **Stent number mean (range)** | 2.5 (1-8) |

Figure SB: **SCAD STEMI comparied with Type I STEMI**. Ejection fraction (a). Infarct size (b). End diastolic (c) and End Systolic (d) voumes.

Figure SC: **Trends in percentage infarct mass after SCAD**. Impact of STEMI status, revascularisation and TIMI flow (a), most proximal vessel involved (b) and extent of dissection (c). Note in panel a data columns are mutually exclusive such that multivessel cases are excluded from other columns and LMS and LAD columns illustrate the most proximal affected segment. Likewise in panel c, patients with recurrent disease are excluded from other columns and patients with both multivessel and multisegment disease are separated from those with only multivessel or multisegment disease. Separate pair-wise statistical comparisons are not shown to avoid multiple testing

Figure SD: **Effect of PHACTR1 genotype on infarct mass in SCAD.** Infarct mass by genotype at the rs9349379 locus on Chromosome 6p24

Figure SE: **Propensity matching.** Effect of PCI complications (a) and conservative versus PCI revascularisation in a propensity matched population (b) on infarct mass in SCAD.

| **Table SB: Univariate predictors of infarction >0% or >10%** | | | | | | | | |
| --- | --- | --- | --- | --- | --- | --- | --- | --- |
| **Covariate** | **Infarction group (%)** | | **Univariate (>0% vs 0%)** | | **Infarction group (%)** | | **Univariate (10+% vs 0-<10%)** | |
|  | **0** | **>0** | **OR (95% CI)** | **P-value** | **0-<10** | **10+** | **OR (95% CI)** | **P-value** |
| **Age at SCAD^** | 44.33 ± 7.98 | 44.62 ± 8.69 | 1.005 (0.966-1.045) | 0.819 | 45.41 ± 8.56 | 41.87 ± 7.40 | 0.946 (0.902-0.992) | 0.0221 |
| **LMS** |  |  |  |  |  |  |  |  |
| No | 55 (38.73) | 87 (61.27) | 1 | 0.6027 | 108 (76.06) | 34 (23.94) | 1 | 0.0835 |
| Yes | 3 (30) | 7 (70) | 1.448 (0.359-5.840) |  | 5 (50) | 5 (50) | 3.147 (0.859-11.527) |  |
| **LAD** |  |  |  |  |  |  |  |  |
| No | 21 (44.68) | 26 (55.32) | 1 | 0.2388 | 42 (89.36) | 5 (10.64) | 1 | 0.0066 |
| Yes | 37 (35.24) | 68 (64.76) | 1.526 (0.756-3.081) |  | 71 (67.62) | 34 (32.38) | 4.079 (1.480-11.239) |  |
| **LCx** |  |  |  |  |  |  |  |  |
| No | 38 (37.25) | 64 (62.75) | 1 | 0.6881 | 76 (74.51) | 26 (25.49) | 1 | 0.9728 |
| Yes | 20 (40) | 30 (60) | 0.867 (0.432-1.739) |  | 37 (74) | 13 (26) | 1.014 (0.468-2.197) |  |
| **RCA** |  |  |  |  |  |  |  |  |
| No | 48 (37.8) | 79 (62.2) | 1 | 0.7994 | 94 (74.02) | 33 (25.98) | 1 | 0.8193 |
| Yes | 10 (40) | 15 (60) | 0.892 (0.371-2.147) |  | 19 (76) | 6 (24) | 0.890 (0.327-2.419) |  |
| **Most severe segment involved** |  |  |  |  |  |  |  |  |
| Proximal | 10 (31.25) | 22 (68.75) | 1.100 (0.386-3.138) | 0.8189 | 19 (59.38) | 13 (40.63) | 1.846 (0.646-5.275) | 0.0485 |
| Mid | 21 (41.18) | 30 (58.82) | 0.748 (0.299-1.874) |  | 38 (74.51) | 13 (25.49) | 0.874 (0.323-2.365) |  |
| Branch | 15 (40.54) | 22 (59.46) | 0.768 (0.288-2.050) |  | 33 (89.19) | 4 (10.81) | 0.310 (0.085-1.128) |  |
| Distal | 12 (36.36) | 21 (63.64) | 1 |  | 24 (72.73) | 9 (27.27) | 1 |  |
| **Multivessel** |  |  |  |  |  |  |  |  |
| No | 51 (40.48) | 75 (59.52) | 1 | 0.2153 | 98 (77.78) | 28 (22.22) | 1 | 0.0388 |
| Yes | 7 (26.92) | 19 (73.08) | 1.809 (0.708-4.619) |  | 15 (57.69) | 11 (42.31) | 2.540 (1.049-6.152) |  |
| **Multisegment** |  |  |  |  |  |  |  |  |
| No | 42 (43.75) | 54 (56.25) | 1 | 0.076 | 77 (80.21) | 19 (19.79) | 1 | 0.0351 |
| Yes | 16 (28.07) | 41 (71.93) | 1.898 (0.935-3.852) |  | 37 (64.91) | 20 (35.09) | 2.222 (1.057-4.670) |  |
| **Initial TIMI** |  |  |  |  |  |  |  |  |
| TIMI 0/1 | 8 (21.62) | 29 (78.38) | 2.733 (1.149-6.497) | 0.0229 | 21 (56.76) | 16 (43.24) | 3.014 (1.361-6.676) | 0.0065 |
| TIMI 2/3 | 50 (43.48) | 65 (56.52) | 1 |  | 92 (80) | 23 (20) | 1 |  |
| **Recurrent** |  |  |  |  |  |  |  |  |
| No | 55 (37.93) | 90 (62.07) | 1 | 0.7756 | 110 (75.86) | 35 (24.14) | 1 | 0.0708 |
| Yes | 3 (37.5) | 5 (62.5) | 0.800 (0.172-3.711) |  | 4 (50) | 4 (50) | 4.152 (0.886-19.459) |  |
| **Presentation** |  |  |  |  |  |  |  |  |
| NSTEMI | 43 (46.74) | 49 (53.26) | 1 | 0.0308 | 77 (83.7) | 15 (16.3) | 1 | 0.0063 |
| STEMI | 12 (23.53) | 39 (76.47) | 2.786 (1.294-5.999) |  | 30 (58.82) | 21 (41.18) | 3.547 (1.616-7.783) |  |
| Cardiac Arrest | 3 (30) | 7 (70) | 1.714 (0.404-7.278) |  | 7 (70) | 3 (30) | 2.533 (0.570-11.268) |  |
| **Pregnancy Status** |  |  |  |  |  |  |  |  |
| Non P-SCAD | 53 (38.41) | 85 (61.59) | 1 | 0.7105 | 105 (76.09) | 33 (23.91) | 1 | 0.1937 |
| P-SCAD | 5 (33.33) | 10 (66.67) | 1.238 (0.401-3.824) |  | 9 (60) | 6 (40) | 2.081 (0.689-6.282) |  |
| **Beighton Score** |  |  |  |  |  |  |  |  |
| <4 | 48 (44.04) | 61 (55.96) | 1 | 0.0228 | 87 (79.82) | 22 (20.18) | 1 | 0.0169 |
| 4+ | 10 (22.73) | 34 (77.27) | 2.543 (1.139-5.677) |  | 27 (61.36) | 17 (38.64) | 2.556 (1.183-5.521) |  |
| **Migraines** |  |  |  |  |  |  |  |  |
| No | 32 (41.56) | 45 (58.44) | 1 | 0.2671 | 61 (79.22) | 16 (20.78) | 1 | 0.1792 |
| Yes | 25 (33.33) | 50 (66.67) | 1.455 (0.751-2.819) |  | 52 (69.33) | 23 (30.67) | 1.659 (0.793-3.470) |  |
| **Mode of revascularisation** |  |  |  |  |  |  |  |  |
| CABG | 1 (14.29) | 6 (85.71) | 3.557 (0.399-31.723) | 0.383 | 4 (57.14) | 3 (42.86) | 4.562 (0.842-24.709) | 0.0273 |
| PCI | 19 (33.93) | 37 (66.07) | 1.386 (0.691-2.778) |  | 36 (64.29) | 20 (35.71) | 2.535 (1.175-5.469) |  |
| Conservative | 38 (42.22) | 52 (57.78) | 1 |  | 74 (82.22) | 16 (17.78) | 1 |  |
| **Any complication** |  |  |  |  |  |  |  |  |
| No | 47 (36.72) | 81 (63.28) | 1 | 0.4813 | 97 (75.78) | 31 (24.22) | 1 | 0.4417 |
| Yes | 11 (44) | 14 (56) | 0.732 (0.307-1.745) |  | 17 (68) | 8 (32) | 1.442 (0.567-3.666) |  |
| **Ethnicity** |  |  |  |  |  |  |  |  |
| Caucasian | 55 (38.19) | 89 (61.81) | 1 | 0.4517 | 108 (75) | 36 (25) | 1 | 0.4433 |
| Non-caucasian | 3 (33.33) | 6 (66.67) | 1.874 (0.365-9.611) |  | 6 (66.67) | 3 (33.33) | 1.784 (0.406-7.840) |  |
| **Smoking Status** |  |  |  |  |  |  |  |  |
| Never | 45 (41.28) | 64 (58.72) | 1 | 0.2312 | 80 (73.39) | 29 (26.61) | 1 | 0.6489 |
| Ever | 13 (29.55) | 31 (70.45) | 1.586 (0.745-3.377) |  | 34 (77.27) | 10 (22.73) | 0.826 (0.362-1.885) |  |
| **Diabetes** |  |  |  |  |  |  |  |  |
| No | 58 (38.16) | 94 (61.84) | NA | NA | 113 (74.34) | 39 (25.66) | NA |  |
| Yes | 0 (0) | 1 (100) |  |  | 1 (100) | 0 (100) |  |  |
| **Hyperlipidemia** |  |  |  |  |  |  |  |  |
| No | 52 (36.88) | 89 (63.12) | 1 | 0.3661 | 103 (73.05) | 38 (26.95) | 1 | 0.1809 |
| Yes | 6 (50) | 6 (50) | 0.580 (0.178-1.892) |  | 11 (91.67) | 1 (8.33) | 0.242 (0.030-1.936) |  |
| **Hypertension** |  |  |  |  |  |  |  |  |
| No | 48 (39.02) | 75 (60.98) | 1 | 0.5779 | 91 (73.98) | 32 (26.02) | 1 | 0.7275 |
| Yes | 10 (33.33) | 20 (66.67) | 1.270 (0.547-2.950) |  | 23 (76.67) | 7 (23.33) | 0.846 (0.331-2.162) |  |
| **CVD History** |  |  |  |  |  |  |  |  |
| No | 47 (40.52) | 69 (59.48) | 1 | 0.2491 | 88 (75.86) | 28 (24.14) | 1 | 0.5332 |
| Yes | 11 (29.73) | 26 (70.27) | 1.599 (0.720-3.551) |  | 26 (70.27) | 11 (29.73) | 1.299 (0.570-2.962) |  |
| **Stroke TIA** |  |  |  |  |  |  |  |  |
| No | 57 (38) | 93 (62) | 1 | 0.8736 | 112 (74.67) | 38 (25.33) | 1 | 0.7653 |
| Yes | 1 (33.33) | 2 (66.67) | 1.217 (0.18-13.736) |  | 2 (66.67) | 1 (33.33) | 1.448 (0.128-16.419) |  |

^mean ± SD; NA analysis not performed due to small numbers.

| **Table SC: Univariate predictors of magnitude of infarction (n=94)** | | | | |
| --- | --- | --- | --- | --- |
| **Correlate** | N | Estimated Mean | 95% CI | Univariate P-value |
| **Age at SCAD^** | 94 | -0.16 | (-0.30, -0.01) | 0.0413 |
| **LMS** |  |  |  |  |
| No | 87 | 9.39 | (8.08, 10.70) | 0.003 |
| Yes | 7 | 16.77 | (12.15, 21.39) |  |
| **LAD** |  |  |  |  |
| No | 26 | 8.06 | (5.59, 10.53) | 0.0797 |
| Yes | 68 | 10.65 | (9.13, 12.18) |  |
| **LCx** |  |  |  |  |
| No | 64 | 9.95 | (8.35, 11.56) | 0.9727 |
| Yes | 30 | 9.9 | (7.56, 12.25) |  |
| **RCA** |  |  |  |  |
| No | 79 | 10.03 | (8.58, 11.47) | 0.7583 |
| Yes | 15 | 9.47 | (6.16, 12.78) |  |
| **Most severe segment involved** |  |  |  |  |
| Proximal | 21 | 12.5 | (9.78, 15.23) | 0.0718 |
| Mid | 30 | 10.37 | (8.09, 12.65) |  |
| Branch | 22 | 7.47 | (4.81, 10.13) |  |
| Distal | 21 | 9.34 | (6.62, 12.06) |  |
| **Multivessel** |  |  |  |  |
| No | 75 | 9.51 | (8.05, 10.98) | 0.205 |
| Yes | 19 | 11.61 | (8.69, 14.53) |  |
| **Multisegment** |  |  |  |  |
| No | 54 | 9.1 | (7.38, 10.83) | 0.1441 |
| Yes | 40 | 11.06 | (9.06, 13.07) |  |
| **Initial TIMI** |  |  |  |  |
| TIMI 0/1 | 29 | 10.8 | (8.43, 13.17) | 0.3853 |
| TIMI 2/3 | 65 | 9.55 | (7.97, 11.13) |  |
| **Recurrent** |  |  |  |  |
| No | 90 | 9.56 | (8.26, 10.86) | 0.0067 |
| Yes | 4 | 18.36 | (12.20, 24.52) |  |
| **Presentation** |  |  |  |  |
| NSTEMI | 49 | 8.48 | (6.69, 10.27) | 0.0669 |
| STEMI | 39 | 11.64 | (9.64, 13.65) |  |
| Cardiac Arrest | 6 | 10.74 | (5.63, 15.85) |  |
| **Pregnancy Status** |  |  |  |  |
| Non P-SCAD | 84 | 9.42 | (8.06, 10.78) | 0.022 |
| P-SCAD | 10 | 14.31 | (10.37, 18.25) |  |
| **Beighton Score** |  |  |  |  |
| <4 | 61 | 9.46 | (7.82, 11.09) | 0.3262 |
| 4+ | 33 | 10.83 | (8.61, 13.05) |  |
| **Migraines** |  |  |  |  |
| No | 44 | 9.78 | (7.85, 11.71) | 0.8265 |
| Yes | 50 | 10.07 | (8.26, 11.89) |  |
| **Mode of revascularisation** |  |  |  |  |
| CABG | 5 | 13.42 | (7.76, 19.07) | 0.1677 |
| PCI | 37 | 10.89 | (8.81, 12.97) |  |
| Conservative | 52 | 8.92 | (7.17, 10.68) |  |
| ^Coefficient (± SE) of regression model | |  |  |  |

| **Table SD: Multivariable predictors of magnitude of infarction in SCAD-survivors** | | | |
| --- | --- | --- | --- |
| **Model** | Estimate | 95% Confidence Interval | P-value |
|  |  |  |  |
| **Multivariable Model 1** |  |  |  |
| **Intercept** | 6.5 | (4.63, 8.37) | <0.0001 |
| **LMS (yes)** | 8.67 | (4.23, 13.12) | 0.0002 |
| **Multivessel (yes)** | 3.25 | (0.24, 6.23) | 0.0344 |
| **Recurrent (yes)** | 8.38 | (2.59, 14.17) | 0.0051 |
| **Cardiac Arrest** | 3.7 | (-1.16, 8.55) | 0.1342 |
| **STEMI** | 3.71 | (1.25, 6.17) | 0.0035 |
|  |  |  |  |
| **Multivariable Model 2 - LMS excluded** |  |  |  |
| **Intercept** | 8.97 | (7.63, 10.30) | <0.0001 |
| **Recurrent (Yes)** | 9.39 | (3.28, 15.51) | 0.003 |
| **Peripartum (P-SCAD)** | 5.34 | (1.34, 9.34) | 0.0095 |
